# Acute beetroot juice supplementation enhances judo-specific performance, explosive power, and muscular strength in recreational adolescent judokas: a randomized crossover trial

**DOI:** 10.3389/fnut.2025.1669799

**Published:** 2025-10-02

**Authors:** Abdullah Demirli, Eda Gökçelik, Abdorreza Eghbal Moghanlou, Muhammed Hüseyin Ocak, Merve Terzi, Emre Yamaner, Müjde Atıcı, Ömer Akyüz, Ali Burak Toy

**Affiliations:** ^1^Faculty of Sport Science, Istanbul University-Cerrahpaşa, Istanbul, Türkiye; ^2^Faculty of Sport Science, Istanbul Aydın University, Istanbul, Türkiye; ^3^Faculty of Sport Science, Istanbul Esenyurt University, Istanbul, Türkiye; ^4^Independent Researcher, Los Angeles, CA, United States; ^5^Faculty of Health Sciences, Istanbul Yeni Yüzyıl University, Istanbul, Türkiye; ^6^Sungurlu Vocational School, Hitit University, Çorum, Türkiye; ^7^Faculty of Sport Sciences, Kahramanmaraş Sütçü İmam University, Kahramanmaraş, Türkiye; ^8^Faculty of Sport Sciences, Bartın University, Bartın, Türkiye; ^9^Faculty of Sport Sciences, Ardahan University, Ardahan, Türkiye

**Keywords:** adolescent athlete, beetroot juice, explosive power, rate of perceived exertion, Special Judo Fitness Test, strength

## Abstract

**Objective:**

Dietary supplementation with beetroot juice (BRJ), rich in nitrate, enhances nitric oxide bioavailability and may positively influence exercise performance. However, the impact of acute BRJ ingestion on judo-specific performance has not been investigated. The aim of this study was to determine the acute effects of BRJ on judo-specific performance, explosive power, back-muscle strength, and handgrip strength in recreational adolescent judokas.

**Methods:**

Thirty-five male adolescent recreational judokas completed a randomized, double-blind, placebo-controlled crossover trial, consuming either 140 mL of BRJ (∼12.8 mmol nitrate) or a placebo, with a 7-day wash-out. In each session, after a 4-min randori, athletes performed the Sargent jump test, back-strength dynamometer pulls, handgrip dynamometer trials, and the Special Judo Fitness Test (SJFT), with a 10-s rest between each performance.

**Results:**

Compared to placebo, BRJ significantly increased explosive jump height (+3%, *p* < 0.05), back-muscle strength, (+3.2%, *p* < 0.01), handgrip strength (+8.4%, *p* < 0.01), and total SJFT throws (+2.4%, *p* < 0.05), while reducing 1-min post-SJFT heart rate (−7.9 b·min^−1^, *p* < 0.001) and improving the SJFT index (−4%, *p* < 0.001). Immediate post-SJFT heart rate and perceived exertion did not differ between conditions (*p* > 0.05).

**Conclusion:**

Acute BRJ is a practical, natural ergogenic aid for adolescent judokas, enhancing explosive power, muscular strength, and judo-specific performance. These findings support the use of nitrate supplementation as an ergogenic aid for young athletes engaged in high-intensity, intermittent exercise activities.

## Introduction

Combat sports such as judo demand exceptional physical capabilities, integrating explosive power, muscular strength, endurance, and sport-specific technical skills within a highly demanding physiological framework. Judo competitions are characterized by intermittent high-intensity efforts interspersed with brief recovery periods, requiring athletes to maintain peak performance across multiple bouts while managing the accumulation of fatigue (1). The metabolic demands of judo training and competition place considerable stress on both aerobic and anaerobic energy systems, with successful performance dependent upon the integration of strength, power, and endurance qualities (2). Understanding how nutritional interventions can enhance these performance determinants represents a critical area of sports science research, particularly given the growing emphasis on evidence-based supplementation strategies in competitive athletics.

The nitrate-nitrite-nitric oxide pathway has emerged as a focal point for exercise performance research, with mounting evidence demonstrating the ergogenic potential of dietary nitrate supplementation. Beetroot juice (BRJ), naturally rich in inorganic nitrates, has gained considerable attention as a performance-enhancing supplement due to its ability to increase plasma nitrite concentrations and subsequently enhance nitric oxide bioavailability (3). The proposed mechanisms underlying these performance benefits include improved endothelial function, oxygen delivery to working muscles, mitochondrial efficiency, and skeletal muscle metabolism and contraction (4–6). These physiological adaptations appear particularly relevant for activities requiring sustained high-intensity efforts, such as those encountered in combat sports.

Extensive research has documented the ergogenic effects of BRJ supplementation across various exercise modalities, though findings have not been universally consistent. Studies investigating endurance performance have generally reported positive outcomes, with BRJ supplementation demonstrating improvements in time-to-exhaustion and exercise economy during cycling and running protocols (7, 8). However, research examining high-intensity, intermittent exercise patterns has yielded more conflicting results. While some investigations have shown enhanced performance in repeated sprint activities and team sports (9), others have reported minimal or no ergogenic effects during similar exercise protocols (10). These discrepancies may reflect differences in study populations, supplementation protocols, exercise testing methodologies, and the specific physiological demands of different sports.

Limited research has specifically examined the effects of BRJ supplementation on combat sport performance, creating a significant knowledge gap in the current literature. de Oliveira et al. (11), for example, demonstrated that a single dose of BR gel accelerated maximal forearm muscle isometric strength recovery 20 min after exhaustive handgrip exercise in recreational combat athletes. Antonietto et al. (12) also reported that BR extract supplementation improved aerobic capacity and taekwondo-specific performance. The few studies that have investigated nitrate supplementation in martial arts contexts have focused primarily on adult practitioners and have not comprehensively evaluated the multifaceted performance demands characteristic of judo competition. Furthermore, the majority of BRJ research has concentrated on single performance outcomes rather than examining the integrated performance profile required for combat sports success (11, 13). This narrow focus limits the practical application of existing research findings to the complex performance requirements of judo athletes.

The potential for BRJ supplementation to enhance multiple performance components simultaneously presents particular relevance for judo athletes, who must integrate explosive power, sustained strength, and technical proficiency throughout competition. Previous research has demonstrated that nitrate supplementation can influence various aspects of muscle function, including force production, power output, and fatigue resistance (14). These diverse physiological effects suggest that BRJ supplementation may provide comprehensive performance benefits that extend beyond single physiological systems, potentially addressing the multifaceted demands of judo competition more effectively than interventions targeting isolated performance components.

The adolescent population represents a particularly important demographic for combat sports research, as this period coincides with critical developmental phases in athletic performance and competitive participation. Adolescent athletes demonstrate unique physiological characteristics that may influence their response to nutritional interventions, including differences in metabolic efficiency, muscle fiber composition, and recovery capacity compared to adult populations (15). Despite these considerations, research examining the effects of BRJ supplementation specifically in adolescent athletes remains scarce. The aim of this study was therefore to evaluate the acute effects of nitrate-rich BRJ on SJFT, explosive power, back-muscle strength, and handgrip strength in recreational adolescent judokas.

## Materials and methods

### Participants

Thirty-five healthy, adolescent male judo athletes (age: 14.2 ± 1.6 years; body mass: 58.7 ± 17.7 kg; body mass index: 21.7 ± 2.7 kg/m^2^; maximal oxygen consumption (V̇O_2_max): 47.4 ± 4.3 mL/kg/min; training age: 4.3 ± 1.2 years) were enrolled in this study. The inclusion criteria were as follows: (a) ≥ 3 years of experience; (b) ≥ 2nd Kyu (blue belt) rank; (c) ≥ 6 months of continuous training; (d) full recovery (at least 3 months) from sports injuries, such as strains and sprains; (e) no history of neuromuscular disorders, smoking, alcohol consumption, or regular medication use. Exclusion criteria included: (a) acute pain/discomfort; (b) failure to complete trials; or (c) protocol non-adherence. Participants trained 3 times per week in judo-specific sessions during the 6 months preceding and throughout the study. All the participants provided written informed consent after being fully informed about the potential risks involved in the experiment.

Sample size estimation was performed with G*Power v3.1.9.7 for a repeated measures ANOVA. Assuming an effect size of 0.25, *α* = 0.05, statistical power = 0.80, two repeated measurements, and a correlation of 0.5 among measures, the analysis indicated that at least 34 participants were required (11). However, 35 athletes were initially enrolled to account for potential participant dropout.

### Study design

**Figure 1:**
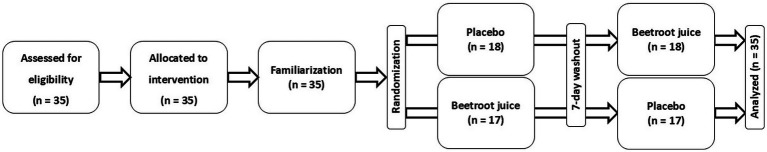
The CONSORT flow diagram and study design.

A randomized, double-blind, placebo-controlled crossover trial was carried out to investigate the acute effects of BRJ on judo-specific performance (Figure 1). The participants were randomly assigned to intake either 140 mL of BRJ (Beet It Sport®, James White Drinks Ltd., UK; ∼12.8 mmol NO₃^−^) or a placebo (140 mL of water with McCormick red food coloring), followed by a seven-day washout period before crossing over to the other trial. The order of the participants was determined through computerized randomization software (Excel Office, Microsoft). Seven days before the main experiments, all participants completed a familiarization session to ensure proper test execution.

### Ethical approval

This study was conducted following the Declaration of Helsinki and received approval from the Ethics Committee for Human Research at Istanbul Aydın University (18/2025) and was registered with the Australian and New Zealand Clinical Trial Registry (ACTRN12625000633482).

### Familiarization

After a 10-min standard warm-up, athletes practiced one or two sets of SJFT, explosive power, back-muscle strength, and handgrip strength tests, as well as being trained to use the Borg rate of perceived exertion (RPE) scale (6–20). Then, they underwent V̇O_2_max measurements by a graded cycling protocol. Briefly, following a 5-min warm-up at 150 W, the workload was increased by 30 W every 3 min until participants reached volitional fatigue. Breath-by-breath respiratory measurements (V̇O₂, V̇CO₂, and ventilation) were obtained via a calibrated PowerCube-Ergo metabolic cart (Ganshorn, Germany). The highest 10-s rolling average of V̇O₂ was designated as V̇O_2_max. Achievement of V̇O_2_max was confirmed when at least three of the following criteria were satisfied: (1) a plateau in V̇O₂ despite further increases in power output, (2) a respiratory exchange ratio exceeding 1.20, (3) attainment of ≥ 90% of age-predicted maximum heart rate, and/or (4) clear signs of exhaustion (16). The session ended with a 5-min cool-down.

### Experimental procedure

We used acute one-dose BRJ supplementation in this study. Participants attended the study site at 7:00 a.m. After consumption of BRJ or placebo in bottles with the same appearance, they remained seated and relaxed for 120 min. A 15-min standardized judo warm-up was then performed, which consisted of 5 min of jogging, followed by 5 min of supervised dynamic exercises (such as walking lunges, heel flicks, high knees, big arm circles, leg swings, and jumping jacks), and ending with 5 min of the ippon-seoi-nage technique uchikomi (repetition training). Following rest for 5 min, young athletes performed a real 4-min randori against an opponent of the same height (± 3 cm), body mass (± 1 kg), and experience (± 1 y). After 30 s rest, a Sargent jump test, back-muscle strength assessment, handgrip strength test, and the SJFT were followed, with 10 s recovery between each measure. Both the randori and SJFT were conducted under the supervision of a qualified judo coach. A 5-min cool-down is executed at the end of the session. Participants were asked to eliminate all high-nitrate foods (e.g., beetroot, celery, spinach, lettuce) from their diets for 48 h preceding each trial. They logged their dietary intake for the 24 h before the initial session and replicated that same diet before the subsequent session. In addition, participants abstained from vigorous exercise for 72 h prior to testing and adhered to regular sleep patterns. All assessments were performed between 7:00 a.m. and 10:00 a.m. after an overnight fast to minimize circadian and nutritional variability. Throughout each trial, standardized verbal encouragement was provided.

### Outcome measures

#### Explosive power

Lower-body explosive power was assessed using the Sargent jump test on a calibrated vertical jump device (Danesh Salar Iranian, Iran). Participants stood sideways to the device, extended one arm to record their maximal standing reach, then performed a countermovement jump—initiating with an arm swing and knee flexion—to touch the highest possible point on the device. Jump height was computed automatically as the difference between standing reach and jump reach.

#### Back-muscle strength

Maximal isometric trunk-extensor strength was assessed using a back-strength dynamometer (Danesh Salar Iranian, Iran). Participants stood on the platform with feet shoulder-width apart, knees flexed to approximately 10°, and hips positioned so that the dynamometer handle aligned with mid-thigh. With the spine erect and head in neutral alignment, they gripped the handle with both hands. Each participant performed one maximal isometric pull—extending the trunk and hips against the immovable handle—holding effort for 3 s.

#### Handgrip strength

Isometric handgrip strength was assessed using a digital handgrip dynamometer (Danesh Salar Iranian, Iran). Participants stood upright with feet shoulder-width apart, held the dynamometer in the dominant hand with the arm at right angles, and the elbow by the side of the body. Without any additional body movement, each subject performed one maximal voluntary contraction, maintaining the squeeze for 3 s.

#### SJFT

It is a practical test developed to assess the physical fitness of judo athletes. The SJFT comprises three bouts of activity—Stage A (15 s) followed by Stages B and C (30 s each)—with 10-s recovery intervals between efforts. Two “ukes” stand 6 m apart; the “tori” (test subject) sprints between them, executing as many ippon-seoi-nage throws as possible during each interval. Heart rate (HR) is recorded immediately upon test completion (HR₀) and again after 1 min of passive recovery (HR₁) using a Polar RS800CX monitor (Finland). The SJFT index is then computed as (HR₀ + HR₁)/total throws (TT), with lower values denoting superior performance (17, 18). Directly after the final effort, participants rate their exertion using the Borg RPE scale.

### Blood sampling and nitrate/nitrite measurement

Venous blood samples were collected by a qualified researcher from the antecubital vein with participants seated, at baseline (pre-supplementation) and immediately before exercise, into EDTA-treated tubes. Samples were centrifuged at 3,500 rpm for 10 min at 4 °C, and the plasma fraction was aliquoted into microcentrifuge tubes and frozen at −80 °C until further analysis. Before assay, plasma was deproteinized by mixing 1:1 (v/v) with ice-cold methanol, followed by centrifugation at 12,000 g for 10 min. The resulting supernatant was diluted in the ENO 20 system’s running buffer (10 mM phosphate, pH 3.0) and injected onto a sulfonated polystyrene divinylbenzene column held at 40 °C. Nitrate (NO₃^−^) and nitrite (NO₂^−^) were separated by ion exclusion HPLC with post-column Griess derivatization (ENO 20; EiCom, Kyoto, Japan). In this configuration, nitrite directly forms an azo dye upon reaction with Griess reagent, whereas nitrate is first reduced to nitrite via a cadmium reduction column before derivatization; both analytes were detected at 540 nm. Quantification was achieved using external calibration curves ranging from 0.1 to 100 μM, with intra- and inter-assay variability below 5% (19). As plasma nitrite concentrations remained below the assay’s 1 μM lower limit of quantification in all participants, only nitrate data are presented.

### Blinding efficacy

At the end of each trial, participants were asked which supplement they thought they had ingested (“BRJ,” “placebo,” or “unsure”) to assess the effectiveness of the blinding (20).

### Statistical analyses

The data are reported as the mean ± standard deviation (SD) and were analyzed using SPSS version 27. Normality was assessed with the Shapiro–Wilk test. The Levene test was used to evaluate the equality of variances. Plasma nitrate concentration was subjected to a two-way repeated-measures analysis of variance (ANOVA) (time × condition). Performance outcomes were analyzed using a paired-samples T test. Effect sizes were calculated using partial eta squared (η_p_^2^) (< 0.1, 0.10–0.24, 0.25–0.39 and ≥ 0.40 for trivial, small, moderate and large effects, respectively) for ANOVA and Cohen’s d (d) (< 0.20, 0.20–0.49, 0.50–0.79, and ≥ 0.8 for trivial, small, moderate and large effects, respectively) for t-test (21). Statistical significance was set at *p* < 0.05.

## Results

All 35 participants completed the study without any dropouts or adverse events and were included in statistical analyses. Participants’ guesses regarding supplement allocation in the two experimental sessions were distributed as follows. In the BRJ trial, young athletes correctly identified the supplement in 27% of cases, incorrectly in 32%, and reported uncertainty in 41%. During the placebo trial, participants guessed correctly 35.3% of the time, incorrectly 29%, and were unsure 35.7%.

**Figure 2:**
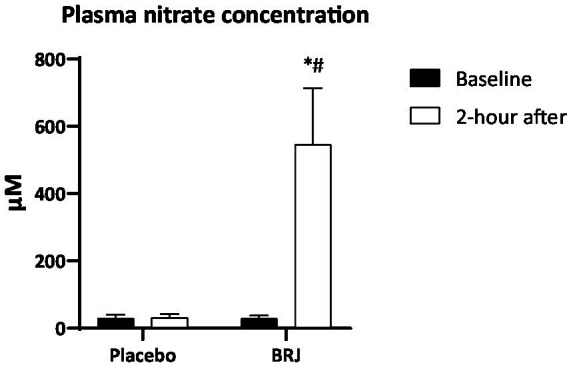
Levels of nitrate in plasma after BRJ and placebo consumption in recreational adolescent judo athletes. ^*^p < 0.001 different from placebo condition. ^#^*p* < 0.001 different from baseline.

**Figure 3:**
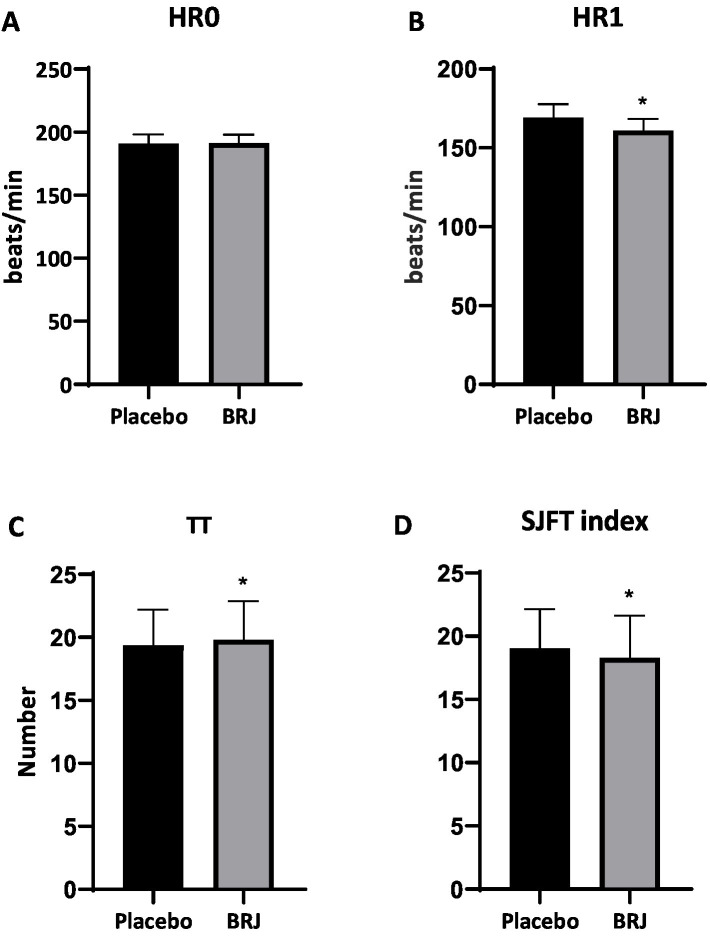
The SJFT outcomes after BRJ and placebo consumption in recreational adolescent judo athletes. HR₀, heart rate immediately upon test completion **(A)**; HR₁ heart rate after 1 min of passive recovery **(B)**; TT, total throws **(C)**; SJFT Index, Special Judo Fitness Test index **(D)**. ^*^p < 0.05 different from placebo condition.

There were significant main effects of time (*p* < 0.001, η_p_^2^ = 0.86) and condition (*p* < 0.001, η_p_^2^ = 0.87), as well as time × condition interaction (*p* < 0.001, η_p_^2^ = 0.86). Consumption of BRJ, but not placebo, increased nitrate concentrations in plasma (Figure 2). BRJ supplementation significantly increased lower-body explosive power (*p* < 0.01, *d* = 0.57), back-muscle strength (*p* < 0.01, *d* = 0.53), and handgrip strength (*p* < 0.05, *d* = 0.35) compared to Placebo (Table 1). There was no significant difference between BRJ and placebo conditions in HR₀ (*p* = 0.331, *d* = 0.16) during SJFT. However, BRJ significantly decreased HR₁ (*p* < 0.001, *d* = 0.85) and SJFT index (*p* < 0.001, *d* = 0.73) and increased TT (*p* < 0.05, *d* = 0.42) compared to placebo (Figure 3). The RPE did not show significant difference between conditions₀ (*p* = 0.476, *d* = 0.12) (Table 1).

**Table 1:** Explosive power, back-muscle strength, handgrip strength, and RPE values after BRJ and placebo consumption in recreational adolescent judo athletes (*n* = 35).

Variables	Placebo	BRJ	Cohen’s *d*
Lower-body explosive power (cm)	43.17 ± 10	44.2 ± 9.4*	*0.57*
Back-muscle strength (kg)	81.23 ± 19.6	83.7 ± 20.2*	*0.53*
Handgrip strength (kg)	28.2 ± 12.1	29.9 ± 12.5*	*0.35*
RPE	17.3 ± 1.6	17.1 ± 1.9	*0.12*

Data are represented in mean ± SD. BRJ, beetroot juice; RPE, rating of perceived exertion. ^*^ *p* < 0.05 different from placebo condition.

## Discussion

The application of BRJ supplementation to combat sports represents a relatively underexplored area of research, despite the theoretical rationale for its potential benefits. The intermittent nature of judo, characterized by explosive techniques separated by periods of gripping and tactical positioning, creates unique metabolic demands that may respond favorably to nitrate supplementation. Previous research has demonstrated that nitric oxide plays important roles in muscle contractility, fatigue resistance, and recovery between exercise bouts (22). These mechanisms suggest that BRJ supplementation could theoretically enhance the multiple performance components required for judo success, including explosive power generation, sustained strength output, and recovery between technical actions. The results of this study demonstrated that acute BRJ ingestion improved lower-body explosive power, back-muscle strength, handgrip strength, and SJFT performance following a real randori in adolescents.

The SJFT represents a validated and sport-specific assessment tool that evaluates the integrated performance capabilities required for judo success. This test protocol incorporates technical skill execution, anaerobic power, and fatigue resistance within a standardized framework that closely mimics the demands of actual judo competition (23). The utilization of sport-specific testing protocols provides enhanced ecological validity compared to generic exercise assessments, offering more meaningful insights into the practical applications of nutritional interventions for combat sport athletes. The improvement in judo-specific performance following BRJ supplementation, as evidenced by increased TT and reduced HR₁ and SJFT index in this study, represents a novel finding in combat sports research (Figure 3).

The observed increases in explosive jump power and muscular strength following BRJ consumption (Table 1) align with several previous investigations examining nitrate supplementation in strength and power activities. Coggan et al. (14) reported improvements in knee extensor speed and power following acute BRJ (140 mL, ∼12.8 mmol nitrate) intake in healthy adults. Similarly, Jodra et al. (24) showed that acute BRJ (140 mL, ∼6.4 mmol nitrate) supplementation improved pre-exercise tension, 30-s Wingate test performance, and lowered post-exercise muscular RPE. Antonietto et al. (12) reported that BR extract (1 g) supplementation improved aerobic capacity and taekwondo-specific performance. It was also shown that a single dose of BR gel (∼12.8 mmol nitrate) accelerated maximal forearm muscle isometric strength recovery 20 min after exhaustive handgrip exercise in recreational combat athletes (11). In addition, Benjamim et al. (25) demonstrated that BRJ extract (600 mg) acutely improved cardiovascular and autonomic recovery after exercise, supporting our findings of reduced HR₁ in SJFT.

The performance enhancements observed in our study can be attributed to the well-established nitrate-nitrite-nitric oxide pathway. Upon consumption, dietary nitrate is reduced to nitrite by oral bacteria, subsequently converted to nitric oxide in tissues with low oxygen availability and acidic conditions (26), precisely the environment created during intense exercise. First, NO can modulate calcium handling within muscle fibers, potentially improving excitation-contraction coupling efficiency (27). Second, NO may enhance muscle fiber recruitment and synchronization during maximal contractions, leading to greater force production (28). Third, the vasodilatory effects of NO improve muscle blood flow and oxygen delivery, supporting energy metabolism during high-intensity efforts as well as improving between trials recovery (29). The reduction in heart rate recovery following the SJFT, while immediate post-exercise heart rate remained unchanged, suggests improved cardiovascular efficiency rather than altered peak cardiovascular stress. This pattern indicates that BRJ supplementation may enhance parasympathetic recovery mechanisms post-exercise, possibly through NO’s effects on autonomic nervous system regulation and improved muscle metabolic efficiency. Given that explosive power, maximal strength, and judo-specific performance predominantly rely on the PCr energy system, acute BRJ intake likely lowers the PCr cost of force production during randori (30) and accelerates PCr resynthesis between bouts (31) thereby enhancing force recovery and subsequent performance.

The present investigation demonstrates that acute BRJ supplementation significantly enhances multidimensional performance parameters in recreational young judokas, providing compelling evidence for the ergogenic efficacy of dietary nitrate across diverse physiological domains. These findings substantially advance our understanding of nitrate-mediated performance enhancement in anaerobic, high-intensity exercise modalities characteristic of combat sports. The maintenance of perceived exertion levels despite improved performance indicates that athletes can achieve greater work outputs at similar subjective intensity levels. This finding has important practical implications, suggesting that nitrate supplementation allows athletes to train at higher intensities without experiencing proportional increases in perceived effort.

### Limitations

Several limitations should be considered when interpreting the results of this study. The acute supplementation protocol, while practically relevant, does not address potential chronic adaptations to nitrate supplementation. Additionally, the study population consisted of recreational adolescent judokas, and results may differ in elite athletes or other age groups. Furthermore, we overlooked genetic differences that could potentially modulate exercise responses (32). Finally, we did not conduct direct measurements of muscle oxygenation, fiber recruitment, or phosphocreatine kinetics, which could be considered another limitation of this study.

## Conclusion

This investigation provides robust evidence that acute one-dose BRJ supplementation enhances multiple aspects of physical performance relevant to judo and combat sports. The observed improvements in explosive power, muscular strength, and sport-specific performance likely result from enhanced nitric oxide bioavailability and its subsequent effects on muscle contractile function and cardiovascular efficiency. These findings support the use of nitrate supplementation as an ergogenic aid for young athletes engaged in high-intensity, intermittent exercise activities.

## Data Availability

The raw data supporting the conclusions of this article will be made available by the authors, without undue reservation.
